# Supplementary material for: Improving Photocatalytic Degradation Activity of Organic Pollutant by Sn4+ Doping of Anatase TiO2 Hierarchical Nanospheres with Dominant {001} Facets
Source: Nanomaterials (Basel). 2019 Nov 12;9(11):1603. doi: 10.3390/nano9111603 (PMC6915639; doi:10.3390/nano9111603)
Supplement: Supplementary file 1 [file nanomaterials-09-01603-s001.pdf]

Supplementary Materials: Improving Photocatalytic Degradation Activity of Organic Pollutant by Sn^{4+} Doping of Anatase TiO_2 Hierarchical Nanospheres with Dominant {001} Facets

Meiling Sun[1], Weichong Kong[1], Yunlong Zhao[1], Xiaolin Liu[1], Jingyue Xuan[1], Yunyan Liu[1], Fuchao Jia[1], Guangchao Yin[1], Jun Wang[1,*] and Junkai Zhang[2,*]

[1] School of Physics and Optoelectronic Engineering, Shandong University of Technology, Zibo, 255000, China

[2] Key Laboratory of Functional Materials Physics and Chemistry of the Ministry of Education, Jilin Normal University, Siping, 136000, China

* Correspondence: junwang1819@163.com (J. W.); zjk8688@126.com (J. Z.)

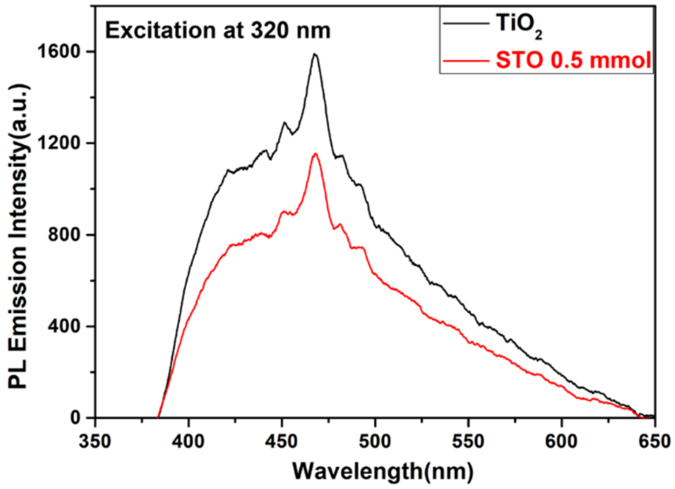

Figure S1. PL spectra of TiO_2 (24 h) and STO (0.5 mmol) hierarchical nanospheres.

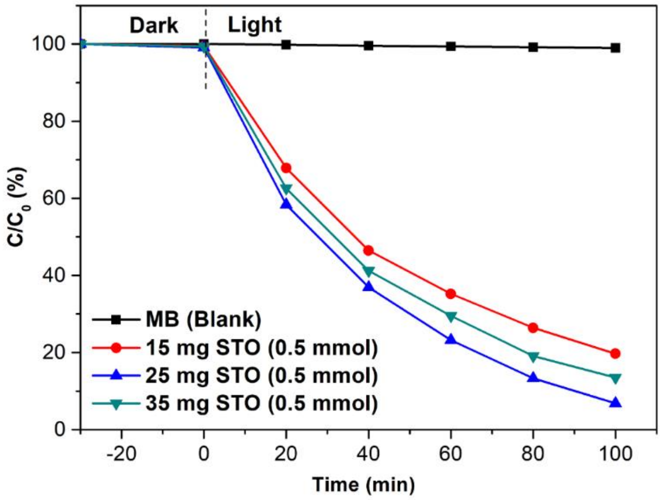

Figure S2. Photocatalytic degradation of MB with STO (0.5 mmol) hierarchical nanospheres at various amounts (15, 25 and 35 mg).
